# Cannabis use in different mental disorders: a descriptive study in a psychiatric hospital

**DOI:** 10.1192/j.eurpsy.2023.733

**Published:** 2023-07-19

**Authors:** B. Samso, A. López Fariña, C. González Navarro, L. Morado San Segundo, A. Bilbao Idarraga, U. López Puentes, R. F. Lopez Brokate, T. Ruiz de Azua Aspizua, U. Ortega Pozas, C. Arán Cisneros, E. Garnica de Cos, I. Alonso Salas

**Affiliations:** Hospital de Zamudio, Bilbao, Spain

## Abstract

**Introduction:**

In the last decade, the prevalence of THC use is increasing among adolescents and adults. There is also strong evidence to suggest that cannabis use is associated with psychiatric comorbidities. The strongest evidence is found between cannabis use and psychotic disorder. However, the literature shows that those who have used cannabis in the past or for a large part of their lives are at higher risk of mood disorders, anxiety, personality disorder or other drug use than those who do not use cannabis in a harmful way.

**Objectives:**

To provide an overview of the association between cannabis use and the different mental pathologies presented by the patients admitted during the study period. To describe the prevalence of THC use in the study according to the mental pathology presented by the patient.

**Methods:**

A retrospective observational descriptive study was developed for 3 months, of all patients admitted to the acute unit of the psychiatric hospital. No exclusion criteria were included.

**Results:**

During the period of study 172 patients were admitted to the hospital, classified according to the main diagnosis we have: 49 patients suffer from schizophrenia, 26 bipolar affective disorder, 20 with depressive disorder, 20 with personality disorder, 19 with substance use disorder, 18 with other unspecified disorders and 20 patients with no known previous diagnosis. The prevalence of THC use in the study sample according to diagnosis, would be schizophrenia 16%, Bipolar affective disorder 19%, Depressive disorder 5%, Personality disorder 45%, Substance use disorder 21%, Unspecified disorders 11% and patients with no known previous diagnosis 10%.

**Conclusions:**

The results obtained in the study in terms of THC use are in agreement with those obtained in the literature. In our study, we observed that cannabis use is associated with psychotic disorders as well as with mood, personality and substance abuse disorders. Given that the frequency of use has increased and there is a strong association with different comorbid psychiatric diagnoses, guidance on modifications in medication strategies might be necessary.

**Disclosure of Interest:**

None Declared

